# Automatic Chessboard Detection for Intrinsic and Extrinsic Camera Parameter Calibration

**DOI:** 10.3390/s100302027

**Published:** 2010-03-15

**Authors:** Arturo de la Escalera, Jose María Armingol

**Affiliations:** Grupo de Sistemas Inteligentes, Universidad Carlos III de Madrid, Avda de la Universidad 30, 28911, Leganés, Madrid, Spain; E-Mail: armingol@ing.uc3m.es

**Keywords:** camera calibration, chessboard detection, pattern recognition, double Hough transform

## Abstract

There are increasing applications that require precise calibration of cameras to perform accurate measurements on objects located within images, and an automatic algorithm would reduce this time consuming calibration procedure. The method proposed in this article uses a pattern similar to that of a chess board, which is found automatically in each image, when no information regarding the number of rows or columns is supplied to aid its detection. This is carried out by means of a combined analysis of two Hough transforms, image corners and invariant properties of the perspective transformation. Comparative analysis with more commonly used algorithms demonstrate the viability of the algorithm proposed, as a valuable tool for camera calibration.

## 1. Introduction

**Figure 1.**
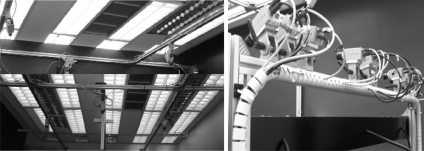
Distribution of cameras in the VISION project.

When a Computer Vision system is designed, a series of parameters must always be considered, which are decisive to the best possible appearance of objects in images with respect to posterior analysis algorithms. The focal length, *f*, allows the elements that are being searched for, to be seen in the image with an adequate size for analysis. Lenses with sufficient quality should be used so that the objects are not deformed in the image. In regard to the positioning of the camera, this is performed so that the space within which the objects are located is perceived in the best possible way. For many applications, these precautionary measures are sufficient. However the calibration of the intrinsic and extrinsic parameters of a vision system, which is fundamental for Computer Vision algorithms, are required to extract three-dimensional information from an image, or a sequence of images, or a system that must establish the relation between two or more cameras. The Project VISION: New Generation Video Communications [1] has motivated the research work presented in this paper. This project involves the use of more than 40 cameras (Figure 1), which are all distributed inside a video-conference room, and are used to perform: stereo reconstruction (18 cameras), Visual Hull (18 cameras) and object tracking (4–6 cameras). These main objectives require the correction of the distortion presented by each of the cameras; finding the relation between images which are captured from different cameras, and the knowledge of the geometric position with respect to the other cameras and the external reference system for each camera. Since there is a large volume of camera equipment being used, it is advisable that the calibration process is fast.

The values that are to be obtained are the horizontal and vertical focal distances, the coordinates of the optical center, the distortion of the lens, and the position and orientation of the camera with respect to the global coordinate system.

This article is laid out in the following way: the next section deals with a description of the advantages of having an automatic calibration system, and the state-of-the-art for automatic detection of calibration points is presented. This is followed by a description of the algorithm that is proposed in this article. Subsequent to this, the next section describes the localization methods for both the main corners and the lines of a chess board pattern separately; after, a description of the combination of this data to determine the points of the pattern is presented. Finally, a series of comparative results using the proposed method compared to more commonly used algorithms is made available, where these results clearly demonstrate the viability of the algorithm proposed.

## 2. Related Work

In general, calibration techniques involve the analysis in images of the projection of a series of characteristic points, which are inherent features of an object whose three-dimensional coordinates are known with a high level of precision. To achieve this, a sequence of images on which the calibration pattern has been captured is made available. Within this pattern, a series of characteristic points are located, which give vital information on the appearance and the distance between the points to be defined. Once these points and their two-dimensional coordinates have been extracted from the sequence of images, the relation between each point of the pattern is found for the series in all the sequences. These are taken as the input for the calibration algorithm, which is used to provide both intrinsic and extrinsic parameters of the camera.

Over the past years, many different types of calibration patterns have been used. The first patterns were of a three-dimensional nature [2], however, several algorithms have been developed that detect one-dimensional patterns such as various points situated on the same line [3,4] or vanishing lines [5,6], but most commonly patterns are dimensional [7]. This is primarily due to the fact that plane calibration patterns are economically effective and their construction is straightforward, given that laser printers are regarded as precision printing devices. These advantages suit the exactness that the majority of image analysis applications demand. The first patterns were formed from matrices of circles or black squares on a white background. However, these patterns were gradually abandoned since the center of gravity of the projections in the image did not coincide with the center of the object due to pattern deformations introduced by the perspective. For this reason, more recently patterns with a similar appearance to that of a chess board are used; here the alternating clear and dark zones favor the detection of the corners of elements on the board.

The above-mentioned calibration method [7] is regarded as one of the most popular techniques, not only due to its precision but also to the fact that it has been introduced into one of the toolboxes used in Matlab [8], DLR Camera Calibration Toolbox [9] and in the libraries of OpenCv developed by Intel [10]. The method uses a range of images where the pattern is detected and the coordinates for each one of the corners is found. The calculation is based on homographies between the two-dimensional pattern and its projection in the image. The implementation in Matlab asks the user to define the area of the image where the pattern is and to give the number of columns and rows of the pattern. The operation of the OpenCv algorithm is based on information supplied by the user, which provides the relation between the points.

The majority of research work in the area of calibration has been centered on the identification of the parameters using robust algorithms, however less attention has been paid to determining the characteristic points that are used as the inputs of the algorithms. The user is left with the more routine and mechanical task of locating the coordinates of the points in the images. The aforementioned task, which was acceptable when no effective calibration algorithms were available, has at the present time a series of disadvantages. These are:

- Since the process is mechanical, users commonly do not pay sufficient attention to the process, and as a result it is subject to errors that are difficult to detect.
- The number of applications is constantly growing and there are many fields of research that use a large number of cameras, thus emphasizing the need for more automatic calibration processes.
- Although the calibration of the camera intrinsic parameters is only carried out once, this is not the case for the extrinsic parameters, which may change on various occasions until the definitive configuration is obtained.
- Up until now, the steps required for the correct calibration process have been carried out by trained and experienced personnel who are familiar with the selection of adequate positions on the patterns, but this situation has to change if such applications are to be performed by users with no specific training in Computer Vision.

Recently, there has been much more research in this area of automatic determination of the calibration points that arise in patterns from a sequence of images taken at different positions. One option which has been considered is that each element in the pattern has a unique code, which helps in the detection and classification process. This method has been used by [11], where instead of using plain squares the use of specially labeled squares is proposed that are automatically defined, which is similar to the Datamatrix method. In [12], a circular element is used that has an internal point code. Other circular patterns are used by [13]. The main difference here is that the annulus of the pattern is coded, so that each circle may be distinguished from others.

Within the group of algorithms where all the elements of the pattern are similar, work has been carried out by [14] where the pattern designed by [15] is used; this consists of a matrix of squares that are not in contact. A smaller clear square is located within the interior of two of the dark squares; these are used to determine the upper left hand corner of the pattern. The edges, which take on a square format, are detected. After rejecting possible candidates that are not sufficiently compact or convex, graph matching is used to detect the pattern.

The rest of the methods use the chess board pattern, which was first presented by [7]. In [16], a chess board pattern is used, where three circles have been added to indicate the orientation of the pattern. First, the corners are located using the Harris detector [17] and these are organized using Delaunay triangulation; this method assures that the triangles are always formed by the closest points. These are grouped together in quadrilateral and after in rows and columns. The method fails when the pattern is too oblique with respect to the camera and when there is a lot of perspective. The work presented by [18] makes use of five patterns formed from two triangles, which are joined at the vertices; an automatic search for these patterns is performed as opposed to the user determining the zone where the board is located. This is carried out using normalized correlation with diverse perspective deformed models. In [19], a chess board pattern is used, where the corners of the image are detected and determine which are due to the intersection between the white and black squares. To complete the differentiation process the system must find two groups of lines, which correspond to the rows and columns of the board. This is carried out by detecting the two most important vanishing points of the image. In [20], they also start the detection process using corner detection. Once these points are located, their mean is obtained and the search of the board starts. This is done by placing the points around the first one and by calculating the main directions by analyzing the gradient. [21] has perfected the algorithm by adding vanishing retro-projection points of the results to fine tune the calculations. In [22], the corners of an image are first obtained. Each one is taken as the center of a circle, and some symmetric constraints of a chessboard pattern are applied: the edges along the border line and the black and white sequence on the chessboard. The grouping step is based on the angles and the property of a cross-ratio.

The recognition of a chessboard using only the detection of its corners is possible if the number of rows and columns is known as well as the area where the chessboard is located as the referenced toolboxes do, but it is very complex if this information is not known and the area is not marked, due to the huge amount of corners an image can have. The recognition using only the lines is possible if the images do not have geometrical distortion and line detection was perfect. The two points with more line intersections would be the clue to find the two families of lines of the chessboard. But images have a distortion, and because of this the edges between the files and columns of the patter are not straight lines and the Hough transform is discrete. Therefore, not all the lines can be detected perfectly. Because of that, the intersection of the lines is not at only two points and this consequence is stronger when the pattern is almost perpendicular to the camera. For these reasons, according to the method proposed in this article, the set of lines are determined from the periodic pattern, which appears in the Hough transform. Since many optical systems have significant geometric distortion, the lines of the pattern are curved in the image and the intersection of these lines does not coincide with the points of the pattern. With this in mind, the previously described analysis is combined with corner detection, on one hand, to validate the lines that are part of the pattern with respect to rest of the objects, and on the other, to determine with sub-pixel precision the true position of the points.

## 3. Board Detection

### 3.1. Corner detection

**Figure 2.**
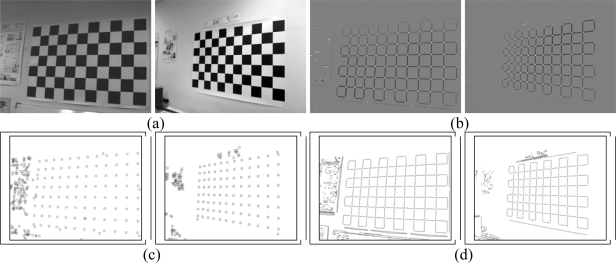
Harris corner detector (a) Original images (b) Harris operator (c) corner detection (d) edges.

The Harris corner detector is widely used to find these features in images. This technique may also be used to find the initial points required for the Hough transform, as its response is positive for corners, almost nil for the uniform zones and is negative for the edges. Once the corner detector has been applied (Figure 2b) a threshold level is set for the image to obtain the corners. On all of the objects of this image a dimensional analysis is performed, which rejects blobs that are too small or too big. From the points remaining their centers of gravity are obtained (Figure 2c). This is one of the steps that define the chess board pattern.

### 3.2. Hough Transform

Further information used is the intersection between the lines of the image. To do this the Hough transform is used based on the points that were detected as edges from the Harris image (Figure 2d).

(1) $$\begin{array}{c} \rho_{1}=x\;\text{cos}\;\theta_{1}+y\;\text{sin}\;\theta_{1}\;\left(\rho_{1}\in \left[-\rho_{1\text{max}},\;\rho_{1\text{max}}\right],\theta_{1}\in \left[-\frac{\pi }{2},\frac{\pi }{2}\right]\right)\\ \rho_{1\text{max}}=\frac{\sqrt{N^{2}+M^{2}}}{2} \end{array}$$

where (*x*,*y*) are the coordinates of the detected edge points and *N* and *M* are the horizontal and vertical dimensions of the image.

**Figure 3.**
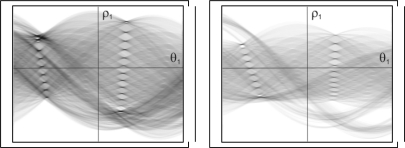
Hough transform of the edge image.

The results obtained from the two images shown in Figure 2, may be observed in Figure 3. Here it can be seen how the maximums of the transform are distributed spatially along a line located from the upper to lower part of the image. These maximums are used as the input values for the second Hough transform, which is used to indicate the set of lines in the image.

In order to obtain the sets of vertical and horizontal lines of the chessboard a series of precautions must be taken into consideration. The first is to decide whether to take a fixed or variable number of maximums, whereby these will depend on the values obtained from the transform. In both cases it involves choosing all lines that form the pattern, but without taking an excessive amount of lines that are not part of the calibration pattern; this will reduce the complexity of the posterior analysis, as can be seen below.

**Figure 4.**
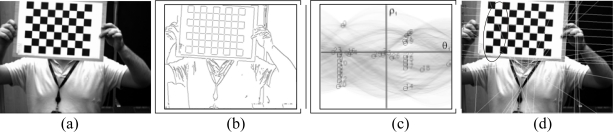
Results from taking a fixed number of lines. (a) Original image (b) edge image (c) Hough transform with its 36 maximums (d) detected lines.

**Figure 5.**
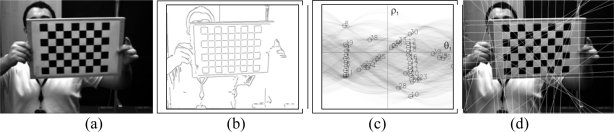
Results from taking a variable number of lines. (a) Original image (b) edge image (c) Hough transform with the maximums (d) detected lines

From Figure 4, it can be seen that the complete set of lines from the pattern have not been detected. The board has 18 lines (taking into account both horizontal and vertical lines) and the algorithm has searched for twice the amount of lines, but still has been unable to detect all of them. The area of the image where the lines have not been detected is indicated (Figure 4d). This technique depends on the number of squares within the pattern, which is information the users do not have to give in our method, and therefore there may exist on occasions an excess or shortage of them. Therefore, a more effective method is to take a variable number for the amount of lines to be located in each image. This implies that a variable number of maximums have to be detected. If the detection criterion is based on a preset threshold level, as can be seen in Figure 5, this determines an excessive number of lines, which will lead to increased complexity in the posterior analysis. This technique fails as it detects maximums that are located in high but uniform zones of the Hough transform, whereas the lines of interest should be determined due to their local maximum nature, which is distinguishable due to their surrounding values.

**Figure 6.**
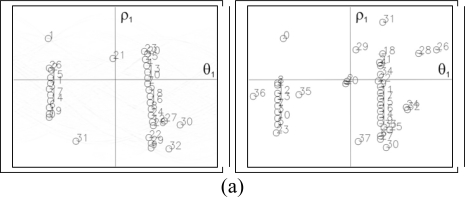

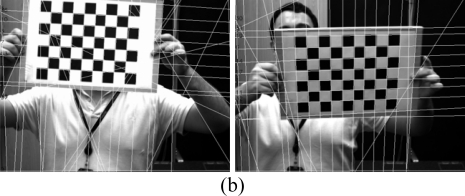
Lines detected using the local maximum search. (a) Local maximums (b) Detected Lines.

To find these points the “top hat” morphological transform is used. This operation is carried out in the following way. A first opening morphological transform is performed on the image and the result is subtracted from the original image. Observing Figure 6, it is seen that the prominent maximums are those that correspond to the local maximums that are required for detection of the pattern. The number of lines is variable and corresponds to those maximums of the morphological transform which are greater than the pre-set value.

### 3.3. Determination of the set of lines

The amount of the detected points which follow the line distribution is observed in Figure 6a. These correspond to those lines where the cut-off point is the same and constitute a similar set of lines. To detect these, a second Hough transform is calculated with the (*ρ*_1_, *θ*_1_) points as the inputs.

(2) $$\begin{array}{c} \rho_{2}=\theta_{1}\;\text{cos}\;\theta_{2}+\rho_{1}\;\text{sin}\;\theta_{2}\;\left(\rho_{2}\in \left[-\rho_{2\text{max}},\;\rho_{2\text{max}}\right],\theta_{2}\in \left[-\frac{\pi }{2},\frac{\pi }{2}\right]\right)\\ \rho_{2\text{max}}=\frac{\sqrt{4\rho_{1\text{max}}^{2}+\pi^{2}}}{2} \end{array}$$

**Figure 7.**
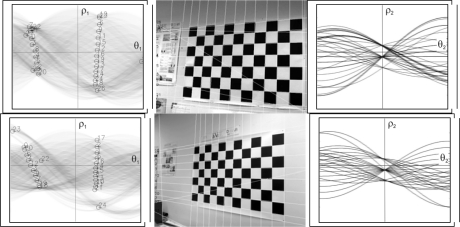
Detection of the set of lines. Maximums of the Hough transform (left column), images with the lines detected (middle column) and the second Hough transform (right column).

As can be seen in Figure 7, the second Hough transform displays the maximum points, which correspond to the set of lines located in the image.

Up to a maximum of three sets of similar lines are sought in the second Hough transform. For each one of these sets, the maximum value is found and compared to the threshold value; if it is greater than this value the set of lines is thus considered to be part of the pattern. In addition, it must be considered that the values of *ρ* may be either positive or negative when using the Hough transform. Once at least two (out of three) maximums are found, the next step is to determine which are the two principle sets of lines and which of the lines belong to each set. The most intuitive solution to perform this task is to calculate the distance of each of the lines (*ρ*_1_,*θ*_1_) to the set of lines (*ρ*_2_, *θ*_2_)

(3) $$\mathrm{dist}=\left|\theta_{1}\;\text{cos}\;\theta_{2}+\rho_{1}\;\text{sin}\;\theta_{2}-\rho_{2}\right|$$

**Figure 8.**
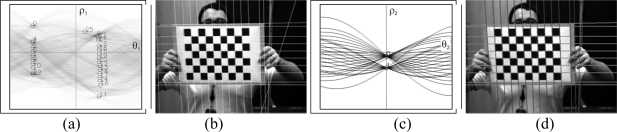
Initial detection of the set of lines.

**Figure 9.**
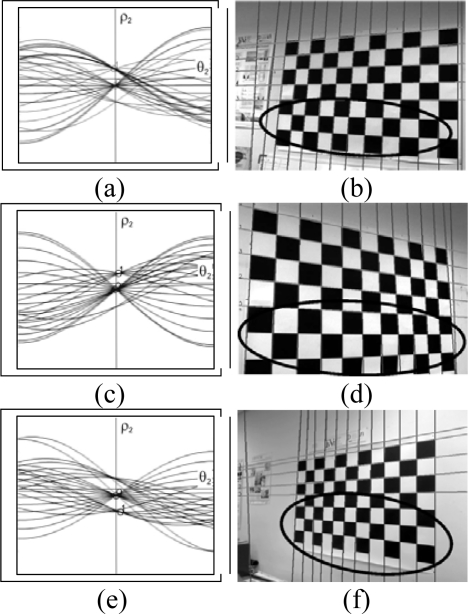
(a-c-e) sets of lines (b-d-f) lines whose distance is small according to (3).

The results using this analysis can be observed in Figure 8, in which a simple case is presented where the pattern is almost perpendicular to the camera. The lines that are detected can be seen as well as how the line that is not compatible with the rest of them is filtered. However, if only the distance given by (3) is considered, numerous errors occur (see Figure 9b-d-f, in which the area of the image where no lines were detected is marked).

**Figure 10.**
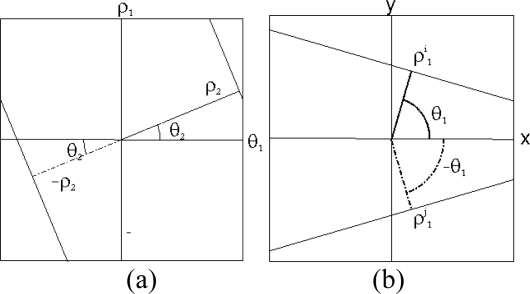
(a) Periodic character of the Hough transform (b) Convergent lines case.

**Figure 11.**
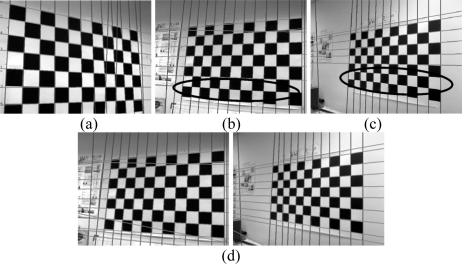
Detection of sets of lines (a) correct detection (b-c) erronous detection (d) correct detections.

These errors occur as the periodic nature of the Hough transform has not been taken into account in the previous analysis (Figure 10a). In this analysis, the angle of the lines is taken to be between $\theta_{1}\in \left[-\frac{\pi }{2},\frac{\pi }{2}\right]$ but in other implementations it has been taken to be between *θ*_1_ ∈ [0, π], where the results are similar but shifted to the right. Thus, a series of lines will be taken (*ρ*_1_, *θ*_1_), which are aligned, but given the interval where *θ*_1_ is defined, they are not found to be represented by the same values of (*ρ*_2_, *θ*_2_) but by (−*ρ*_2_, *θ*_2_). As can be observed in Figure 11a–c, more lines are detected. However it can also be seen that all the lines forming the patterns in Figure 11b and c have not been located. This is principally due to the fact that the pattern is not only formed from parallel lines but also by convergent lines; as a result these lines must also be associated to the detected set of lines.

It may be concluded from this analysis that the complete set of lines forming the pattern is composed of both parallel and convergent lines. In Figure 10b, the case is presented where convergent lines form part of the image of the pattern, *i.e.*, the lines parallel to the line *i* will all have similar angles *θ*_1_ and will also have the values obtained from the second Hough transform (*ρ*_2_, *θ*_2_) and (−*ρ*_2_, *θ*_2_) associated with them. When considering the line *j*, which is convergent, this line has a similar value of *θ*_1_ but is negative, thus the lines parallel to it will be closer to the values (*ρ*_2_, −*θ*_2_) and (−*ρ*_2_, −*θ*_2_). To determine the complete set of lines, four different distances are calculated; if for any of the lines they are less than a pre-set value these lines are then associated to the set of lines (Figure 11d).

### 3.4. Pattern localization

To locate the pattern in the image, both the horizontal and vertical sets of lines must be determined. To do this the three previous sets of lines are taken and the one with the smallest number of lines associated with it is disregarded. From the two remaining sets, one will correspond to the horizontal lines and the other to the vertical lines. If the average value of the angle is calculated as:

(4) $$\theta_{i}=\frac{\sum_{k=1}^{n_{i}}\left|\theta_{1}^{i}\right|}{n_{i}}\;\;\;\;\;i=(1,2)$$

the set of lines with the smallest value will correspond to the vertical lines.

The lines belonging to the chess board pattern must comply with the fact that the crossing points between the horizontal and vertical lines are close to one of the corners, which were found with the Harris corner detector (Figure 2c). Thus, for each crossover point between the lines, all detected corners are analyzed, the closest corner is chosen and the crossover point is considered to be valid if the distance is less than the threshold value. This corner is then removed from the list. Upon finishing the process for all the crossover points, each line—both horizontal and vertical—has a series of intersections associated with it, which are considered as valid.

The following step involves two different processes. The first process removes the lines with a small number of crossover points, as these correspond to remote lines, which do not form part of the pattern but are located in the background. The number of valid crossing points is again calculated and followed by the same process of elimination of the lines containing few crossover points.

When a line is eliminated this changes the common crossover points, therefore these two processes are performed iteratively until no more lines are eliminated.

Once the previously described processes reach the stage where no lines are removed, the second process detects the set of lines having the same number of crossover points. These lines correspond to the chess board pattern. Then, the average value of the crossover points is calculated for the horizontal and vertical lines. The lines, both horizontal and vertical, where the amount of crossover points is less than the average value, are eliminated; the process is repeated until none of the horizontal or vertical lines are eliminated.

**Figure 12.**
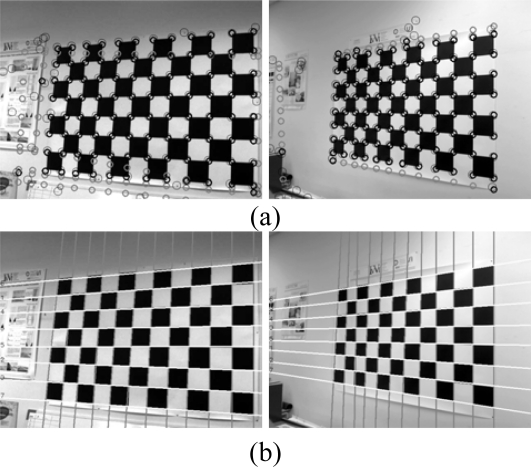
(a) Pairing up of the crossover points, corners and (b) detected lines.

The two groups of lines remaining are thus the lines of the chess board pattern. Different results using this elimination process are presented in Figure 12. The circles with the thick black lines are the chess board corners where information from the sets of lines and corners coincide, the thick grey lines correspond to the intersections of lines which do not correspond to any corner, and the thin black circles are the rest of the corners in the image.

Once a series of lines has been obtained, the next task is to determine which of those lines actually correspond to the board pattern, as the situation may arise where a line is parallel to the axis of the board but does not belong to the pattern. To eliminate such lines the chess board pattern is used, whose number of rows and columns is unknown, however its squares have known dimensions; using this information all the lines that do not comply with this cross-ratio can be removed, since this value is invariant in the projection.

The cross-ratio of four aligned points, A, B, C and D is expressed as:

(5) $$\text{RD}(\text{ABCD})=\frac{\bar{\text{AC}}}{\bar{\text{BC}}}\frac{\bar{\text{AD}}}{\bar{\text{BD}}}$$

Since the points have the same vertical coordinate, it may be assumed that the four points are consecutive and belong to a horizontal line:

(6) $$\begin{array}{cc} \begin{array}{cc} A=(x,y) & B=(x+\Delta x,y)\\ C=(x+2\Delta x,y) & D=(x+3\Delta x,y) \end{array} & \text{RD}(\text{ABCD})=\frac{2\Delta x}{\Delta x}\frac{3\Delta x}{2\Delta x}=3 \end{array}$$

where Δ*x* refers to the dimensions of the square. It is important to point out that the invariant value does not depend on this value.

The lines are ordered from left to right and from top to bottom. The double ratio for the first four vertical lines on the left hand side of the pattern is calculated, if the result is within the 5% error margin of the value obtained in (6) it is considered as valid. If not, the first line is rejected and the fifth line is added and the value is recalculated. This process is continued until the first four lines are obtained that satisfy the criteria, providing the lines on the left hand side of the pattern. In a similar way, the last four vertical lines on the right side of the pattern are obtained where the double ratio is again calculated and the lines are accepted or rejected depending on the result. Following the same method the horizontal lines are also obtained. Once this process is finished the outline of the complete chess board pattern is obtained.

The last step of the algorithm is to eliminate the external lines of the board, so that the system only detects the interior area of the pattern. This process is carried out for two reasons:

1. The external lines that outline the board consist of lines where only half of the squares provide points for the Hough transform, these lines are not always detected. If a robust detection system is required involving different positions and conditions an element whose detection is uncertain should not be added. Even though this problem can be solved the following reason advises against its use.
2. The detection of the board serves for the detection of corners, the edge of this pattern is where the corners are most poorly defined; this is because there are no alternating black and white squares in both directions, which is the case for the rest of the corners. For this reason they are not considered as good points to be used in the calibration process.

**Figure 13.**
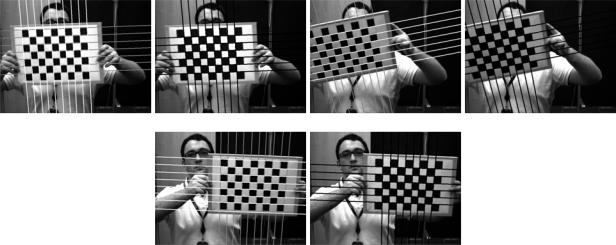
Final line filter.

To confirm if these lines should be removed from the detection algorithm, the value obtained from the Hough transform is observed and compared to the width of the pattern obtained. If it does not exceed the preset threshold level this indicates that a discontinuous line has been detected. This process is repeated for the four edges of the pattern. Results from the final detection process may be observed in Figure 13.

**Figure 14.**
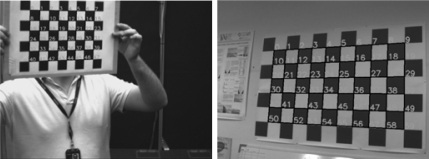
Input points of the calibration algorithm.

Finally, due to the geometric distortion, the crossover points between the lines will not coincide with the real location of the corners of the chessboard. For this reason, the corners are searched within the crossover points’ surroundings, and are used in the final calibration. (Figure 14).

The system is calibrated by the implementation of Zhang’s algorithm, which has been developed within OpenCV. It is a function that has as inputs the corners detected in the sequence of images and as output the intrinsic parameters of the camera.

**Figure 15.**
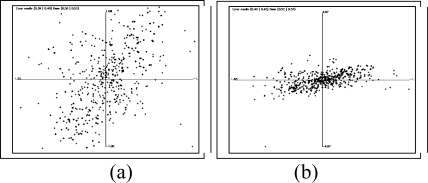
Errors between the theoretical projection and the real projection of the points of the pattern in the image for (a) the initial calibration (b) refined results.

Once these values have been obtained the translation and orientation respect to the chessboard for every image of the sequence can be calculated, this will help to refine the intrinsic parameters. To perform this process, the points of the pattern are projected onto the image. The error between the real value for the point projection and the theoretical is calculated (Figure 15a), from which the average value is obtained, *μ_e_*, and the standard deviation, *σ_e_*. All points where the error is outside the range of (*μ_e_* − 3*σ_e_*,*μ_e_* + 3*σ_e_*) are rejected. The system is calibrated again with the points lying within this interval. If the error introduced is calculated again using this data (Figure 15b) it is observed to be reduced.

### 3.5. Calibration of the extrinsic parameters

**Figure 16.**
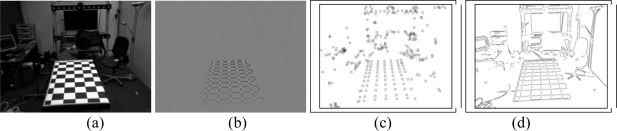

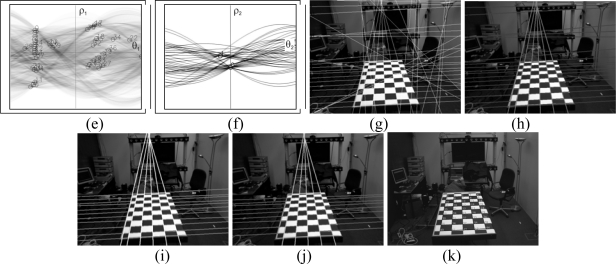
(a) original image (b) Harris operator (c) detected corners (d) edges of the objects (e) Hough transform (f) Hough transform of the principle lines (g) principle line (h) sets of lines (i) lines which are coherent with the corners detected (j) final lines (k) board.

The system described does not serve only for the calibration of the intrinsic parameters, it also allows calculation of the position and orientation of the camera with respect to a global reference. As can be observed in Figure 16 a chessboard is also used, although much larger, which is positioned in a known position, rotation and translation, in respect to the room axis of references. Almost the same algorithm is applied for the detection of the chessboard. The only difference is an additional first step: as the intrinsic parameters have been calibrated before and are known, the geometrical distortion can be corrected in the image. The system detects the chessboard and situates the camera within the global reference system. This case is much more complex than that of the calibration of the intrinsic parameters. As the pattern is further away and does not occupy the majority of the image, more objects are found in the background. Here it is observed that the number of corners is many.

## 4. Results

To demonstrate the feasibility of the results obtained from the proposed system, they were compared to detection techniques that are already well established. This analysis was carried out for both the detection of the board pattern and the calibration values.

The OpenCV method first performs a thresholding, then followed by an erosion process, which is used to break the connectivity of the squares; after this it finds their number and position in the image. Since information in regards to the number of rows and columns is supplied, the erosion process is repeated as many times as is necessary to break the connectivity and find the corresponding numbers. Next, the squares are brought back together to form the board pattern. The number of points must be passed through each calibration and the pattern must be completely visible in the image, as is opposite to the technique proposed in this article.

The results of the calibration with the proposed method were compared to those obtained with the Bouguet Matlab Toolbox for three sequences, which are presented in Table 1. It can be observed that the results are similar and the errors committed are within the same range of margins, the main difference found when using this technique is that the method proposed here does not require each corner of the chess board pattern to be marked, neither the knowledge of the number of rows and columns. The image dimensions for the sequences S1 and S2 are 640 × 480 pixels and 1,024 × 768 for S3.

**Figure 17.**
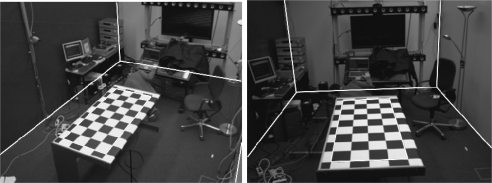
Drawing from two cameras of the outline of the room.

Concerning the extrinsic parameters, an image is presented in Figure 17 where the distortion is eliminated from the image The corners of the room, which have been projected using the intrinsic and extrinsic parameters, are superimposed onto the image. Clear agreement is seen with the results.

The algorithm has been tested on two different equipments with images of 1,024 × 768 pixels and takes 1.9 seconds in an Intel Core2 Duo Mobile Processor at 1.66 GHz with 2 GB of RAM and 1.1 seconds in an Intel Core2 Duo at 3.00 GHz with 3.25 GB of RAM.

## 5. Conclusions

In this article, an automatic camera calibration method has been presented. This is achieved by using an algorithm proposed in this article to detect an artificial pattern located within an image. The only information available in the calibration pattern used is that it is composed of a chess board configuration. The algorithm is capable of determining the number of rows and columns of the pattern. Using different examples, it has been shown that the algorithm is a valuable tool for camera calibration and that its use will make this task less tedious, allowing the process to be easily performed by non-specialized personnel.

## Figures and Tables

**Table 1.** Comparison of the calibration values between the Toolbox of Matlab of Bouguet and this method.

		**fx (px)**	**fy (px)**	**cx (px)**	**cy (px)**	**Radial distortion**		**Tangential distortion**		**Error x (px)**	**Error y (px)**

						**k1 10^−3^**	**k2 10^−3^**	**p1 10^−3^**	**p2 10^−3^**		
S1	Bouguet	821.1	812.8	327.4	234.6	−248.5	214.9	2.5	−5.1	0.50	0.36
	Our method	820.6	812.4	327.4	234.7	−247.0	210.9	2.5	−5.1	0.39	0.40
S2	Bouguet	514.3	515.9	322.1	243.0	23.9	−157.5	3.1	1.5	0.30	0.17
	Our method	517.3	518.8	320.6	239.8	3.1	−54.4	1.4	0.7	0.24	0.23
S3	Bouguet	2371.6	2421.8	326.3	389.8	−35.7	684.7	−3.96	−57.5	1.17	0.31
	Our method	2440.0	2493.0	328.2	387.9	28.0	334.9	−4.2	−59.5	0.93	0.39
